# FK506 Attenuated Pilocarpine-Induced Epilepsy by Reducing Inflammation in Rats

**DOI:** 10.3389/fneur.2019.00971

**Published:** 2019-09-12

**Authors:** Aihua Wang, Zhihua Si, Xiaolin Li, Lu Lu, Yongli Pan, Jinzhi Liu

**Affiliations:** ^1^Department of Neurology, Shandong Provincial Qianfoshan Hospital, The First Hospital Affiliated With Shandong First Medical University, Jinan, China; ^2^Department of Neurology, Shandong Provincial Qianfoshan Hospital, Shandong University, Jinan, China

**Keywords:** FK506, status epilepsy, inflammatory response, radical content, neuroprotective

## Abstract

**Background:** The status epilepticus (SE) is accompanied by a local inflammatory response and many oxygen free radicals. FK506 is an effective immunosuppressive agent with neuroprotective and neurotrophic effects, however, whether it can inhibit the inflammatory response and attenuate epilepsy remains unclear.

**Objective:** This study aims to clarify the effect of FK506 on inflammatory response in rats with epilepsy.

**Methods:** A total of 180 rats were randomly and equally divided into the control group, epilepsy group, and FK506 group. The rat SE model in the epilepsy group and FK506 group was induced by lithium chloride combined with pilocarpine. In the FK506 group, FK506 was given before the injection of pilocarpine. The control group was given the same volume of saline. Then the effect of FK506 on epilepsy in rats and the changes of inflammatory factors and free radicals in hippocampus were examined using hematoxylin and eosin (HE) staining, immunohistochemistry, quantitative real-time polymerase chain reaction (qRT-PCR), and western blotting.

**Results:** FK506 ameliorated the course of pilocarpine-induced epilepsy and the neuronal loss in the rat hippocampus after SE. FK506 reduced the increased content of nitric oxide (NO), superoxide dismutase (SOD), and malondialdehyde (MDA) in the hippocampus after SE. Besides, FK506 also significantly reduced the levels of factors involved in inflammatory response such as vascular cell adhesion molecule-1 (VCAM-1), intercellular adhesion molecule-1 (ICAM-1), tumor necrosis factor-α (TNF-α), and Protein Kinase C δ (PKCδ) that rise after epilepsy.

**Conclusion:** FK506 ameliorated the course of pilocarpine-induced epilepsy, significantly reduced free radical content, and inhibited the expression of inflammatory factors, which provided a theoretical basis for the application of FK506 in the treatment of epilepsy.

## Introduction

Epilepsy is a neurological disorder that is characterized by abnormal brain activity, causing detrimental effects on cognitive, psychological, and social behaviors of patients (1). It is associated with many neurological changes, such as astrocyte activation and neuronal necrosis (2, 3). Status epilepticus (SE) refers to persistent or recurrent seizures due to a failure of seizure termination mechanisms (2, 4). The guidelines from the International League Against Epilepsy (ILAE) suggest that 10-min duration could be used as a time point for which focal SE can be defined and 60-min duration for which long-term consequences might emerge (5). The hippocampus, located between the cerebral thalamus and the medial temporal lobes, is part of the limbic system and plays a key role in learning and memory. Studies have shown that hippocampal sclerosis in patients with medial temporal lobe epilepsy (MTLE) is associated with neuronal loss and significant gliosis (3, 6). The previous study demonstrated that neuronal loss and markable gliosis are associated with hippocampal sclerosis in patients with MTLE (6). In various animal models of epilepsy, loss and degeneration of pyramidal neurons are common in all areas of the hippocampus (7–9). Changes in the hippocampus, including reactive gliosis, are reported to be the cause of cognitive impairment induced by epilepsy (9, 10).

However, the mechanism of epilepsy is very complex. Animal models have emphasized the involvement of inflammatory mediators in seizure susceptibility and epileptogenesis (11, 12). Robust and general inflammatory responses in the brain lower the seizure threshold, enhance neuronal excitability, increase blood-brain barrier permeability, and promote epileptogenesis. Some studies have revealed that there are some changes in the brain inflammatory mediators in patients with epilepsy (13–15); anti-inflammatory effects of increased steroid hormone by adrenocorticotropic hormone (ACTH) treatment could play a crucial role in the suppression of refractory epilepsy in West syndrome (16); intravenous immunoglobulin can suppress seizures in some types of intractable epilepsy, partially through reducing cytokines and suppressing astrocyte activation (17, 18).

FK506, a calcineurin inhibitor, is a potent immunosuppressant for clinical prevention and treatment of allograft rejection (19–21). It exerts its immunosuppressive effect by binding to FK506 binding protein 12 (21, 22). Studies have shown that FK506 has neuroprotective and neurotrophic effects (23, 24). As reported, FK506 has neuroprotective effects in a mouse model of cerebral ischemia and seizures, but the specific neurons targeted by FK506 and the underlying mechanism of its neuroprotective effects remain unclear. Gant et al. found that FK506 can prevent kainic acid-induced seizures and inhibit the germination of mossy fibers in the hippocampus, thereby exerting neuroprotective effects (25). Moreover, it has been reported that FK506 can attenuate inflammation in a rat model for spinal cord injury (26). FK506 also can reduce neuroinflammation in an α-synuclein-based Parkinson's disease model (27). However, whether FK506 can inhibit the local inflammatory response in the injured brain tissue to inhibit epileptic state remains unknown.

In this study, we explored the changes of inflammatory factors in the SE rats treated with FK506 to investigate the effect and mechanism of FK506 on epilepsy.

## Materials and Methods

### Ethics Statement

This study was approved by the Ethics Committee of Qianfoshan Hospital Affiliated to Shandong University, China [2016; Ethical approval number (S018)]. All procedures and protocols were performed according to our institutional guidelines.

### Model Establishment

A total of 180 healthy male Wistar rats, with body weights of 250–280 g were purchased from the Animal Center of Shandong University Medical College. The rats were randomly and equally divided into the control group, epilepsy group, and FK506 group. Pilocarpine was used to establish the SE model. The rats in the epilepsy group and FK506 group were intraperitoneally injected with 3 mol/(L·kg) lithium chloride (Sigma, USA); after 18–20 h, 30 mg/kg of pilocarpine (Sigma, USA) was given intraperitoneally. Moreover, in the FK506 group, 0.5 mg/kg FK506 was intraperitoneally injected at 24 and 1 h before the injection of pilocarpine. The control group was given an intraperitoneal injection of the same volume of saline. The rat seizure symptoms were graded based on the Racine grading criteria (1972). Level 0: No response; Grade I: Facial clonus (blinking, moving, rhythmic chewing, etc.); Level II: Grade I plus rhythmic nod; Grade III: Grade II plus forelimb myoclonus without upright hind limbs; Grade IV: Grade III plus upright hindlimb; Grade V: generalized tonic, a burst of seizures, and loss of position control. One hour after acute SE, 200 mg/kg of chloral hydrate was given intraperitoneally to relieve convulsions to reduce mortality, and survivors with Racine grade IV and above were used for further analysis.

A total of 24 rats in every group were randomly and equally divided into four groups and then subjected to cardiac perfusion at different time points (6, 12, 24 h, 7 d) after SE for immunohistochemistry and hematoxylin and eosin (HE) dying. The remaining 36 in each group were also randomly and equally divided into six groups and sacrificed at 3, 6, 12, 24 h, 3, and 7 d after SE; fresh hippocampal tissues were isolated and stored at −80°C (Figure S1).

Notably, the death rate of the model establishment was about 30%, so the actual number of used rats was more, and the number of successfully modeled rats (grades IV–V) was 180.

### HE Staining

The brain tissue was removed and immediately embedded in OCT (SAKURA, Japan), followed by being frozen in a refrigerator at −80°C. The tissues were placed in a cryostat for 30 min and sectioned into 20-μm pieces. The tissue pieces were fixed with 4% paraformaldehyde for 15 min and rinsed three times with phosphate buffer saline (PBS) for 5 min each time. Then the tissue pieces were stained with hematoxylin for 10 min and eosin for 30 s−1 min, followed by the gradient alcohol dehydration, xylene transparent, and final neutral gum sealing.

### Immunohistochemistry

Rats in each group were perfused at 6, 12, 24 h, 7 d after successful modeling. Rats were anesthetized with chloral hydrate at 350 mg/kg. The chest and abdomen were cut open to expose the heart and liver. The perfusion needle punctured through the left ventricular apex. Then the right atrial appendage was opened, followed by the rapid perfusion of 200 mL heparinized saline and then 200 mL paraformaldehyde (4%) until the rat limbs became straight and the liver turned white. The whole brain was removed, fixed in 4% paraformaldehyde for 12 h, and transferred to 20 and 30% sucrose overnight. The brain tissue was then cut into 10-μm thickness. Immunohistochemical staining was performed using the ABC kit (Thermo Scientific, USA). All procedures were conducted according to the manual instructions. The antibodies used are listed as follows: neuronal nitric oxide synthase (nNOS) (1:1,000, Chemicon), protein kinase C-delta (PKCδ) (1:300, Santa Cruz), vascular cell adhesion molecule-1 (VCAM-1) (1:50, Sigma), intercellular adhesion molecule-1 (ICAM-1) (1:50, Sigma), active caspase-3 (1:10, Chemicon). Notably, all stainings were performed at the same time point.

### Measurement of Oxidative Stress Markers

The whole hippocampal tissue was homogenized in 10 volumes of cold water. Oxidative stress markers were measured in the homogenate, including nitric oxide (NO), superoxide dismutase (SOD), and malondialdehyde (MDA). Measurements were performed using an ultraviolet spectrophotometer (28). For NO measurement, the Griess reaction was used to measure nitrates (29). Briefly, specimens were added to the Griess reagent, followed by the addition of trichloroacetic acid. The product was read at 520 nm. For SOD, the xanthine-xanthine oxidase system generated superoxide radicals and reacted with radical iodonitrotetrazolium (INT) to form colored formazan, which was measured at a wavelength of 505 nm. Determination of MDA levels was based on the color formed with thiobarbituric acid (30), and the product was measured at 532 nm.

### Quantitative Real-Time Polymerase Chain Reaction (qRT-PCR)

Total RNA was extracted using Trizol (TAKARA, USA) according to the instructions of the manufacturer. Then, the concentration and optical density (OD) value were determined. Subsequent experiments were performed on RNA with an OD value between 1.8 and 2.0. Two micrograms of RNA per sample was reverse transcribed into cDNA with a reverse transcription kit, done according to the protocol of the manufacturer (TAKARA, USA). The reaction system for qRT-PCR was prepared following the instruction of kit (TAKARA, USA). The reaction conditions were as follows: pre-denaturation at 95°C for 1 min, denaturation at 95°C for 10 s, annealing, and extension at 60°C for 30 s, 40 cycles in total. Reduced glyceraldehyde-phosphate dehydrogenase (GAPDH) was used as the internal reference of target genes. The expression of genes was calculated using the relative quantitative measurement (2^−ΔΔCt^). The primer sequences are shown below.

VCAM-1:
F 5′-GGAAATGCCACCCTCACCTT-3;
R 5′-CACCTGAGATCCAGGGGAGA-3′.
ICAM-1:
F 5′-GCCTGGGGTTGGAGACTAAC-3′;
R 5′-CTGTCTTCCCCAATGTCGCT-3′.
Tumor necrosis factor (TNF):
F 5′-CATCCGTTCTCTACCCAGCC-3′;
R 5′-AATTCTGAGCCCGGAGTTGG-3′.
PKC:
F 5′-CAAAGGCCGCTTCGAACTCTAC-3′;
R 5′-GGCCATCCTTGTCCAGCATTAC-3′.
GAPDH:
F 5′-GGCACAGTCAAGGCTGAATG-3′;
R 5′-ATGGTGGTGAAGACGCCAGTA-3′.

### Western Blotting

Rat hippocampal tissues were removed and washed once with PBS and lysed with radioimmunoprecipitation assay (RIPA) lysis buffer for 10 min at room temperature. The lysates were centrifuged at 15,000 g for 10 min at 4°C, and 5 X loading buffer was added to the supernatant and mixed. The protein was heated at 95°C for 10 min. Then 40 μg protein was loaded into each lane and separated on 10% sodium dodecyl sulfate-polyacrylamide gel electrophoresis (SDS-PAGE). After electrophoresis, the protein was transferred to a polyvinylidene (PVDF) fluoride membrane. Then, the membrane was blocked with 5% BSA for 1 h at room temperature and then incubated with the primary antibody at 4°C overnight, followed by the incubation with the corresponding secondary antibody (1:5,000) at 37°C for 1 h. Protein bands were detected using X-ray film using an enhanced chemiluminescence system (Nikon). GAPDH was used as a positive control. The band gray values were analyzed using Image J. The antibodies used are listed as follows: nNOS (1:20,000, Chemicon), PKCδ (1:1,000, Santa Cruz), VCAM-1 (1:500 Sigma), ICAM-1 (1:500, Sigma), active caspase-3 (1:200, Chemicon).

### Statistical Analysis

Statistical analysis was performed using the SPSS statistical software package (SPSS 13.0 for Windows; SPSS, Inc., Chicago, IL USA). The data were expressed as mean ± standard deviation (SD). Differences between the two groups were analyzed by the two-tailed Student's *t*-test. The differences between three or more groups were analyzed by one-way analysis of variance, followed by Tukey's honestly significant difference (HSD) *post hoc* test. ^*^P < 0.05 was considered statistically different.

## Results

### FK506 Attenuated Pilocarpine-Induced Seizure in Rats

The rats in the control group did not exhibit any seizure behaviors. Pilocarpine induced-behavioral seizure showed aggravating symptoms in the intensity and duration, which gradually progressed toward status episodes. The FK506-treated group exhibited a longer latency and a smaller percentage at stage IV and V than those of the epilepsy group (Table 1).

**Table 1:** The latency of grade IV seizures and percentages of grade IV and above seizures in rats.

**Group**	**Samples**	**Latency (mean ± SD)**	**Percentage of grade IV seizure**	**Percentage of grade V seizure**
Epilepsy group	60	45.6 ± 8.8	41.7%	58.3%
FK506 group	60	66.1 ± 9.3^*^	71.7%^*^	28.3%^*^

* *P < 0.05 vs. epilepsy group*.

### FK506 Attenuated Neuronal Loss in the Hippocampus of Rats After SE

**Figure 1:**
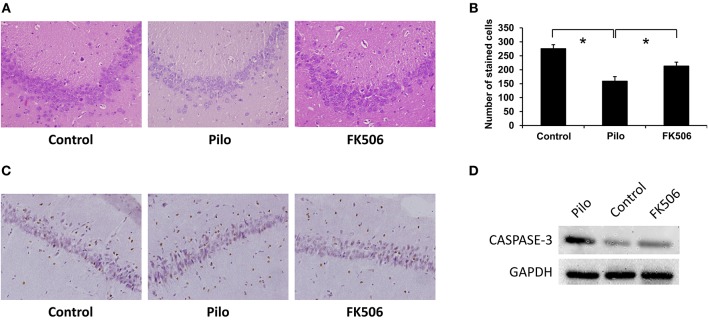
Changes of neuronal loss in the hippocampus of rats in different treatment groups. **(A)** Morphological changes (HE staining) of the hippocampus in different treatment groups after 7 days of modeling. Magnification: 400 X. **(B)** Unbiased quantification of HE stained pyramidal CA3 cells. *P < 0.05. **(C)** Caspase-3 expression in the control group, epilepsy group, and FK506 group after 7 days of modeling detected by immunohistochemistry. Magnification: 400 X. **(D)** Protein expression of Caspase-3 in the control group, epilepsy group, and FK506 group after 7 days of modeling detected by western blotting.

Hematoxylin and eosin (HE) staining can reveal structural changes in neurons of the hippocampus. The results showed that normal living cells have round nuclei and are palely stained. In contrast, neurons in the epilepsy group had nuclear pyknosis. Compared with the control group, the HE-stained cells decreased in the epilepsy group. However, when FK506 was administered prior to pilocarpine injection, the number of HE-stained cells increased after SE, although it did not reach the level of the control group (Figures 1A,B). Moreover, in order to confirm the neuronal apoptosis in the hippocampus, we also examined the expression of apoptosis-related marker caspase-3 at 7 d after onset, which showed that FK506 significantly reduced the expression of caspase-3 (Figures 1C,D), and the neuroprotective effect of FK506 was thus verified.

### FK506 Reduced the Levels of NO, MDA, and SOD in the Hippocampus of Rats After SE

**Figure 2:**
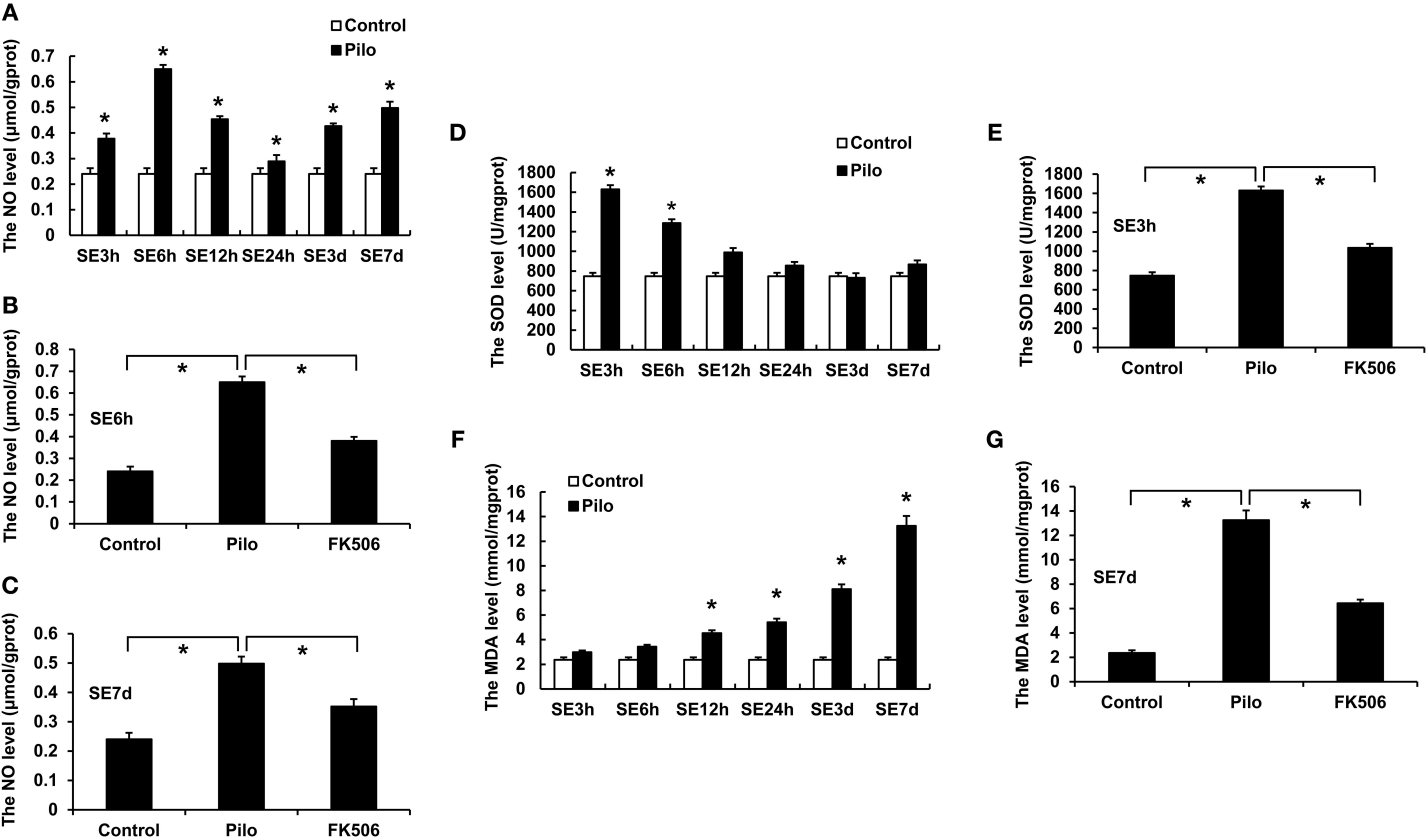
Changes of NO, MDA, and SOD in the hippocampus of rats in different treatment groups. **(A)** Changes of NO at different time points in the control group and the epilepsy group. **(B)** Changes of NO in the control group, epilepsy group, and FK506 group after 6 h of modeling. **(C)** Changes of NO in the control group, epilepsy group, and FK506 group after 7 days of modeling. **(D)** Changes of SOD at different time points in the control group and the epilepsy group. **(E)** Changes of SOD in the control group, epilepsy group, and FK506 group after 3 h of modeling. **(F)** Changes of MDA at different time points in the control group and the epilepsy group. **(G)** Changes of MDA in the control group, epilepsy group, and FK506 group after 7 days of modeling. *P < 0.05.

The level of nitric oxide (NO) in the hippocampus of epileptic rats rose dramatically after SE, reaching the peaks at 6 h (0.652 ± 0.016 μmol/gprot) and 7 d (0.498 ± 0.023 μmol/gprot) (Figure 2A). FK506 can significantly reduce the content of NO both in the acute phase (6 h; 0.384 ± 0.020 μmol/gprot) and late phase (7 d; 0.349 ± 0.024 μmol/gprot) of SE (Figures 2B,C).

Besides, superoxide dismutase (SOD) level increased significantly at 3 h (1,628.7 ± 40.6 13 U/mgprot) after the seizure, while malondialdehyde (MDA) level gradually increased and reached the highest level at 7 d (13.250 ± 0.748 mmol/mgprot) after onset (Figures 2D,F). After the FK506 intervention, the SOD content after the 3-h seizure (1,034.727 ± 38.677 U/mgprot) and the MDA content after the 7-d seizure (6.446 ± 0.299 mmol/mgprot) were significantly reduced (Figures 2E,G).

### FK506 Decreased the mRNA Expression of Inflammatory Factors in the Hippocampus of Rats After SE

**Figure 3:**
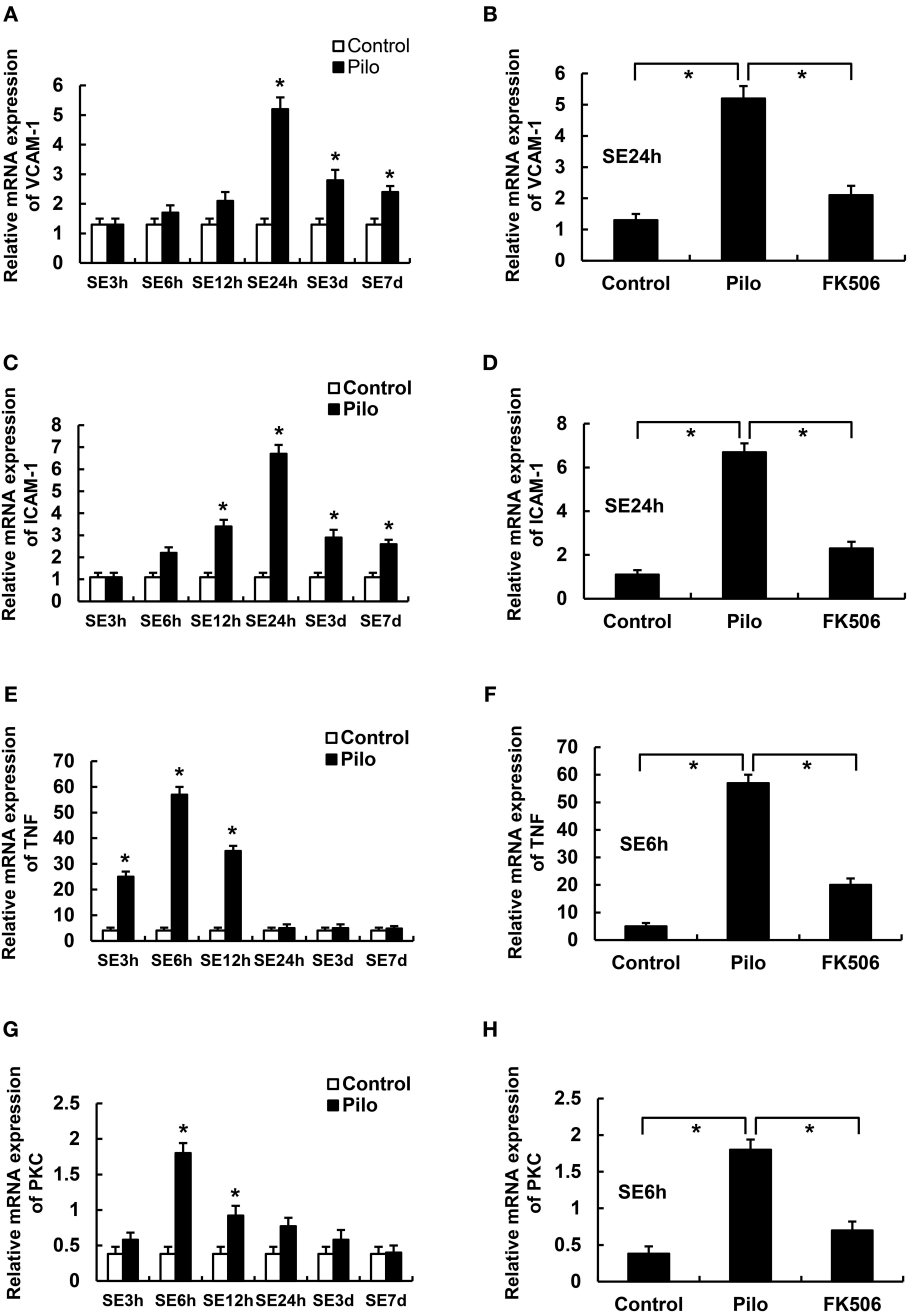
The RNA levels of VCAM-1, ICAM-1, PKCδ, and TNF in the hippocampus of different treatment groups. **(A)** VCAM-1 changes at different time points in the control group and the epilepsy group. **(B)** VCAM-1 changes in the control group, epilepsy group, and FK506 group after 24 h of modeling. **(C)** ICAM-1 changes at different time points in the control group and the epilepsy group. **(D)** ICAM-1 changes in the control group, epilepsy group, and FK506 group after 24 h of modeling. **(E)** TNF-α changes at different time points in the control group and the epilepsy group. **(F)** TNF-α changes in the control group, epilepsy group, and FK506 group after 6 h of modeling. **(G)** PKCδ changes at different time points in the control group and the epilepsy group. **(H)** PKCδ changes in the control group, epilepsy group, and FK506 group after 6 h of modeling. *P < 0.05.

We first examined the mRNA levels of vascular cell adhesion molecule-1 (VCAM-1), intercellular adhesion molecule-1 (ICAM-1), tumor necrosis factor-α (TNF-α), and Protein Kinase C δ (PKCδ) at different time points after SE. The results showed that the VCAM-1 and ICAM-1 mRNA levels in the epilepsy group were highest at 24 h after SE, which were significantly higher than those in the control group (Figures 3A,C). Compared with the control group, PKCδ and TNF-α were significantly increased in the acute phase (6 h) but gradually decreased in the later stage of SE in the epilepsy group (Figures 3E,G). To clarify the effects of FK506 on these inflammatory factors, we examined the expression levels of VCAM-1 and ICAM-1 at 24 h and PKCδ and TNF at the corresponding time points in different groups. It was found that FK506 significantly reduced the mRNA expression of all these inflammatory factors at the above time points (Figures 3B,D,F,H).

### FK506 Decreased the Protein Expression of Inflammatory Factors in the Hippocampus of Rats After SE

**Figure 4:**
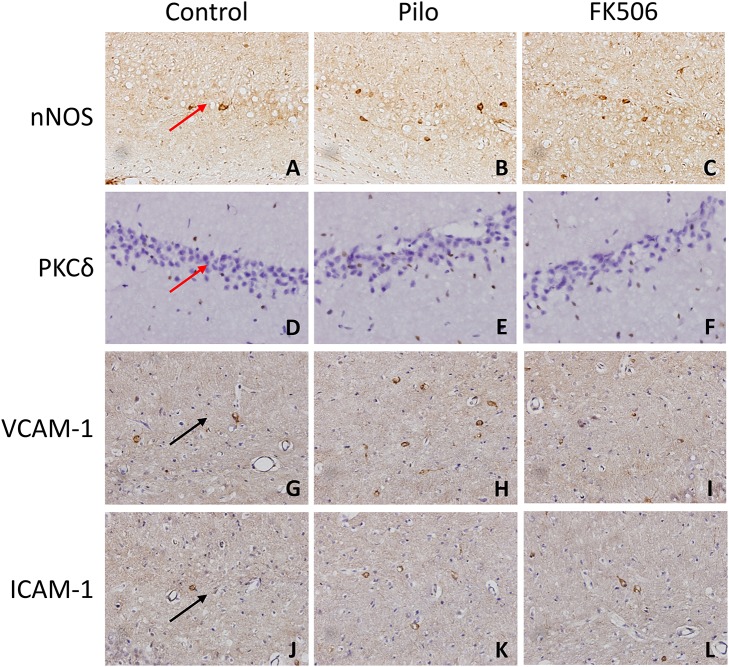
The *in situ* expression of VCAM-1, ICAM-1, PKCδ, NOS, and caspase-3 in the hippocampus of different treatment groups. **(A–C)** NOS expression in the control group, epilepsy group, and FK506 group after 6 h of modeling. **(D–F)** PKCδ expression in the control group, epilepsy group, and FK506 group after 6 h of modeling. **(G–I)** VCAM-1 expression in the control group, epilepsy group, and FK506 group after 24 h of modeling. **(J–L)** ICAM-1 expression in the control group, epilepsy group, and FK506 group after 24 h of modeling. Magnification: 400 X. Red arrow, CA3 region. Black arrow, vascular endothelium.

**Figure 5:**
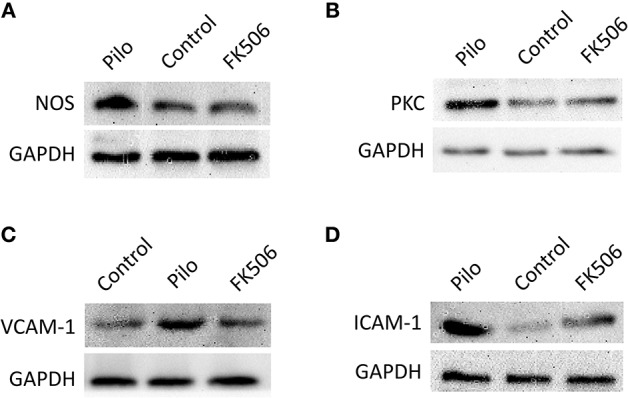
The protein expression of VCAM-1, ICAM-1, PKCδ, and TNF in the hippocampus of different treatment groups. **(A)** Protein expression of NOS in the control group, epilepsy group, and FK506 group after 6 h of modeling. **(B)** Protein expression of PKCδ in the control group, epilepsy group, and FK506 group after 6 h of modeling. **(C)** Protein expression of VCAM-1 in the control group, epilepsy group, and FK506 group after 24 h of modeling. **(D)** Protein expression of ICAM-1 in the control group, epilepsy group, and FK506 group after 24 h of modeling. The internal reference was glyceraldehyde-3-phosphate dehydrogenase (GAPDH).

To further clarify the protein and *in situ* expression of these inflammatory factors in the hippocampus, histochemical staining, and western blot were performed. We obtained the rat hippocampus tissues at the time point when the expression difference was most significant according to the RNA results (VCAM-1 and ICAM-1 at 24 h and PKCδ at 6 h after onset). Compared with the epilepsy group at the corresponding time points, FK506 intervention reduced the protein expression of these inflammatory factors to levels similar to that in the control group (Figures 4D–L, 5B–D). Compared with the epilepsy group, FK506 treatment also significantly reduced the expression of nNOS at 6 h after SE (Figures 4A–C, 5A).

## Discussion

It has been reported that FK506 has neuroprotective and neurotrophic effects (23, 24). The previous study showed that FK506 can prevent kainic acid-induced seizures by inhibiting the production of mossy fibers in the hippocampus (25). In the present study, we observed that the FK506 attenuated pilocarpine-induced seizure and neuronal loss in the hippocampus of rats possibly by reducing the free radical content and inhibiting the expression of inflammatory factors.

The mechanisms underlying the therapeutic effects of FK506 have been extensively studied (31). Except for the induction of immunosuppression through calcineurin inhibition of the NF-AT pathway of T lymphocytes, FK506 increases superantigen-induced T cell apoptosis by decreasing the survival gene (Bcl-XL) expression, possibly making FK506 act as an anti-inflammatory agent via inhibiting activation of the immune system. Local inflammatory responses may be involved in chronic epilepsy as well as SE, leading to persistent tissue damage and increasing the likelihood of seizures and recurrence.

The oxidative stress response is one part of the inflammatory response. NO is a messenger and gas neurotransmitter in the central nervous system, and NOS is the most critical factor in the regulation of NO biosynthesis (32). NO is produced in each reaction process of inflammation and involved in a variety of inflammatory signaling pathways. Our results showed that the NO content and nNOS expression in the hippocampus increased after seizures, indicating that they are associated with seizures. Moreover, FK506 could reduce the content of NO and the expression of nNOS in the hippocampus of epilepsy, thereby reducing inflammation and the damage of NO on brain tissue. Oxygen free radicals are the effector of the inflammatory response, and excess free radicals, in turn, promote the development and persistence of inflammation. The damage of oxygen free radicals on cells is mainly caused by lipid peroxidation of biofilm unsaturated fatty acids. MDA is a metabolite of lipid peroxidation of biofilm unsaturated fatty acids by oxygen free radicals (33). The change in its content indirectly reflects the change in oxygen free radical content in the tissue. Therefore, the level of oxygen free radicals and the strength of lipid peroxidation can be estimated by measuring the MDA content level. SOD is a kind of negatively charged protein that dissociates superoxide anion radical O^2−^ by disproportionation. As a scavenger of free radicals, the SOD content also reflects the number of free radicals. In this study, the MDA and SOD levels in the FK506 intervention group decreased compared with those in the epilepsy group, suggesting that FK506 can reduce the production of free radicals after epilepsy, thereby reducing inflammation and playing a neuroprotective role.

Clinical studies have identified changes in circulating inflammatory cells in patients with epilepsy (34–36). In addition, peripheral inflammatory cells are infiltrated in damaged and epileptic brain tissue (37–39). The area of neuronal death and dysfunction in epilepsy tissue is closely related to the increase in the number of inflammatory cells and the increase in inflammatory factors (11, 34, 40, 41). Spontaneous recurrent seizures result in the chronic expression of VCAM-1, which might affect blood-brain barrier permeability, neuroinflammation, and subsequent seizures (42). ICAM-1 was reported to be correlated with the intensity of seizures (43). The importance of TNF-α-mediated inflammation has also been demonstrated in the development of acute seizures. TNF-α regulates glutamate receptor transport through TNF receptor 1 to induce enhanced excitatory synaptic transmission (44). Besides, PKC overactivity impairs prefrontal cortical regulation of working memory (45) and is associated with both seizure activity and SE. For example, PKC activity blocks neuropeptide Y-mediated inhibition of glutamate release and contributes to the excitability of the hippocampus in SE (46). We found that FK506 not only had direct neuroprotective effects but also inhibited the expression of inflammatory factors VCAM-1, ICAM-1, PKCδ, and TNF-α after seizures, thereby indirectly inhibiting the development of epilepsy.

Moreover, FK506 also impacts the glucocorticoid receptor (GR) signaling pathway by binding to the FK506-binding protein FKBP-52 in the GR complex and thus facilitating the nuclear entry of GR, where they might interact with transcriptional factors that are important regulators of inflammatory responses (e.g., nuclear factor-kB) to enhance the anti-inflammatory effects of steroids. Therefore, in future work, we would examine whether FK506 regulates the activities of transcriptional factors to control the levels of inflammatory factors to attenuate epilepsy.

In conclusion, this study showed that FK506 ameliorated the course of pilocarpine-induced epilepsy and had the neuroprotective effects in the hippocampus of rats after SE. Moreover, FK506 significantly reduced free radical content and inhibited the expression of inflammatory factors, which were closely related to seizure onset. Therefore, FK506 showed good therapeutic effects in the rat epilepsy model, providing a new therapeutic strategy for epilepsy in the future.

## Data Availability

The datasets generated for this study are available on request to the corresponding author.

## Ethics Statement

The animal study was reviewed and approved by the Ethics Committee of Shandong Provincial Qianfoshan Hospital, the First Hospital Affiliated with Shandong First Medical University, China.

## Author Contributions

AW participated in designing the study, interpreted the data, and wrote the manuscript. ZS, XL, LL, and YP performed the experiments, collected the data, and analyzed the data. JL conceived and designed the study and provided critical revisions that are important for the intellectual content. All authors read and approved the final version of the manuscript.

### Conflict of Interest Statement

The authors declare that the research was conducted in the absence of any commercial or financial relationships that could be construed as a potential conflict of interest.

## Supplementary Material

Figure S1 A representative diagram of all steps of the experimental design, the number of animals used in each group/time point studied.
