# Supplementary figures and images for: FK506 Attenuated Pilocarpine-Induced Epilepsy by Reducing Inflammation in Rats
Source: Front Neurol. 2019 Sep 12;10:971. doi: 10.3389/fneur.2019.00971 (PMC6751399; doi:10.3389/fneur.2019.00971)

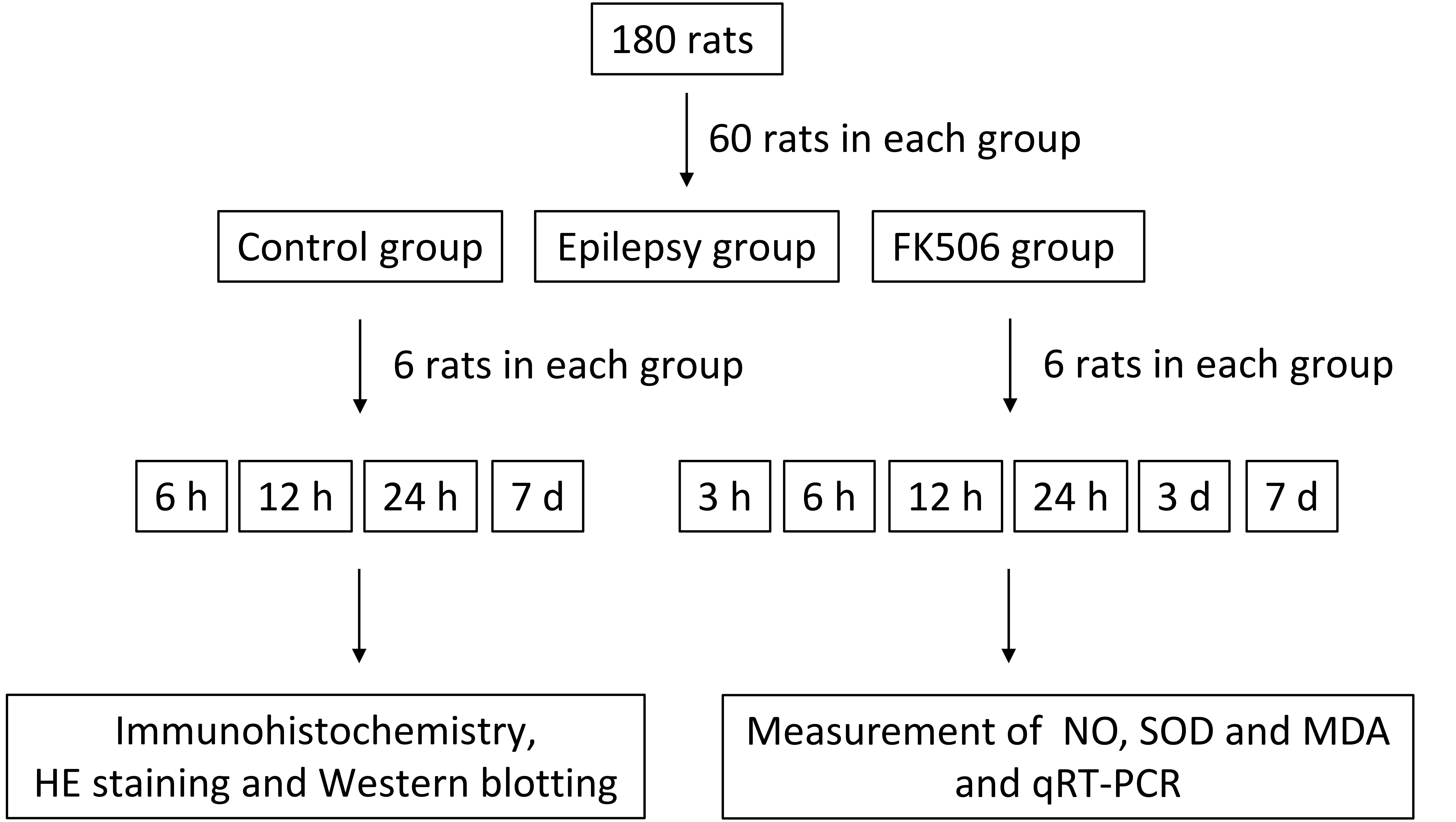

Supplement: Figure S1 — A representative diagram of all steps of the experimental design, the number of animals used in each group/time point studied. [file Image_1.TIF]
